# Sense of Coherence and Perceived Academic Stress Among Nursing Students: A Multicenter Cross-Sectional Study

**DOI:** 10.3390/nursrep15080288

**Published:** 2025-08-08

**Authors:** David Ballester-Ferrando, Esther Cáceres-Malagelada, Carolina Rascón-Hernán, Teresa Botigué, Ana Lavedán, Olga Masot, Dolors Burjalés, Luis González-Osorio, Ximena Osorio-Spuler, Eva Serrat-Graboleda, Concepció Fuentes-Pumarola

**Affiliations:** 1 Department of Nursing, University of Girona, 17003 Girona, Spain; david.ballester@udg.edu (D.B.-F.); esther.caceres@udg.edu (E.C.-M.); carolina.rascon@udg.edu (C.R.-H.); concepcio.fuentes@udg.edu (C.F.-P.); 2 Department of Nursing and Physiotherapy, University of Lleida, 25198 Lleida, Spain; teresa.botigue@udl.cat (T.B.); ana.lavedan@udl.cat (A.L.); olga.masot@udl.cat (O.M.); 3 Nursing Department, Universitat Rovira i Virgili, 43007 Tarragona, Spain; mdolors.burjales@urv.cat; 4 Departamento de Medicina Interna, Oficina de Educación en Ciencias de la Salud, Facultad de Medicina, Universidad de La Frontera, Temuco 4811230, Chile; luis.gonzalez@ufrontera.cl; 5 Departamento de Enfermería, Facultad de Medicina, Universidad de La Frontera, Temuco 4811230, Chile; ximena.osorio@ufrontera.cl; 6 Center of Excellence for Physics and Health Engineering (CFIS), Universidad de La Frontera, Temuco 4811230, Chile

**Keywords:** mental health, nursing students, sense of coherence, stress

## Abstract

**Background:** Nursing students often face high academic and emotional demands, which can negatively affect both their mental health and academic performance. From a salutogenic perspective, the sense of coherence (SOC) is considered a key protective factor in managing stress and fostering resilience. **Objectives**: This study aimed to explore the SOC levels among nursing students and examine their associations with perceived academic stress and sociodemographic variables. **Methods**: A multicenter, cross-sectional, exploratory study was conducted in a sample of 1301 undergraduate nursing students from four universities in Spain and Chile. Participants completed the Orientation to Life Questionnaire, a validated instrument assessing SOC and its three dimensions: comprehensibility, manageability, and meaningfulness. Sociodemographic data and students’ perceived stress in relation to key academic activities were also collected. Descriptive and inferential statistical analyses were performed, including *t*-tests and ANOVA. **Results**: The mean SOC score was 62.65 (SD = 12.36), with no significant differences between universities. Significant associations (*p* < 0.05) were found between SOC scores and age, marital status, academic year, work status, and university entry path, but not with gender or caregiving responsibilities. Students aged ≥29 years and those who were married or working had higher SOC scores. Higher levels of perceived stress in lectures, seminars, clinical practice, group work, and written assignments were significantly associated with lower SOC scores. **Conclusions**: This study’s findings suggest that a stronger SOC is associated with lower perceived academic stress and certain sociodemographic characteristics. Integrating salutogenic approaches into nursing curricula could strengthen students’ SOC, promoting their mental well-being and academic resilience.

## 1. Introduction

Nursing students engage in an academic environment characterized by high cognitive and emotional demands [1], especially during clinical practice [2]. These inherent challenges contribute to psychological distress, depression, and anxiety [3] which, in turn, negatively affect both academic performance [4] and overall mental health and well-being [5,6]. In a study conducted in England involving pre-registration nursing students, the researchers classified stressors into three domains: academic (e.g., examinations, fear of failure during training, workload pressures), clinical (e.g., clinical responsibilities, fear of making mistakes, emotional responses to patient suffering or death, interpersonal dynamics within the healthcare team), and personal/social (e.g., financial concerns, balancing household obligations with academic tasks) [7,8]. Other investigations have reported that the level of stress depends on biopsychosocial characteristics, such as income or age, in addition to academic characteristics [9].

Several studies have demonstrated that the implementation of active learning methodologies yields positive outcomes, notably through enhancing motivation, fostering social skills, deepening content comprehension, and solidifying students’ study habits [10]. However, the literature examining the specific impacts of these methodologies—distinguished by type of activity—on academic stress remains scarce and yields heterogeneous results. For example, Cardozo et al. [11] found that introducing gamification as an active learning strategy not only improves knowledge acquisition, but also facilitates more effective stress management among nursing students. Conversely, Baraz et al. [12] reported that nursing students frequently experience high levels of stress associated with formative activities—particularly during clinical practicums—which may adversely affect both their well-being and academic outcomes.

Antonovsky’s salutogenic paradigm posits the existence of relationships among stress, social class, culture, health, and disease [13]. He observed that certain individuals, despite facing stressful circumstances, maintain or even enhance their health. Within this framework, Antonovsky introduced the sense of coherence (SOC) as an overarching orientation reflecting one’s enduring sense of confidence and perceiving life as structured, predictable, and explicable [14,15]. From the salutogenic perspective, protective factors—such as resilience and a strong sense of coherence—gain prominence, enabling students to appraise academic situations as comprehensible, manageable, and meaningful [16,17]. Indeed, evidence suggests that students with higher SOC scores develop more adaptive coping strategies when confronted with academic and professional stressors [18,19].

On the other hand, the absence of adequate coping mechanisms to meet the inherent demands of nursing studies—and the subsequent professional responsibilities—can have detrimental consequences on mental health and overall well-being [20], which may compromise the future professional performance of an individual [21].

Therefore, the aim of this study was to explore the SOC levels among nursing students and examine their associations with perceived academic stress and sociodemographic variables. This analysis can provide valuable information for the identification of students at risk, guide preventive interventions, and promote both well-being and academic performance.

## 2. Materials and Methods

### 2.1. Study Design

This is a multicenter, cross-sectional, exploratory study that adheres to the STROBE guidelines (see Supplementary Materials) for reporting observational research [22].

### 2.2. Population and Sample

The study population comprised nursing undergraduates from all four academic years at three public universities in Spain (University of Lleida, Rovira i Virgili University, and University of Girona) and one university in Chile (University of La Frontera) (N = 2055). A convenience sampling approach was used, accessing classrooms with a confidence level of 99% and a margin of error of 3%. Based on these parameters, a minimum sample size of n = 972 participants was required. All undergraduate students were offered the chance to voluntarily participate in the study, and those recruited were selected through convenience sampling. The eligibility criteria included nursing students over 17 years old, consent to participate in this study, understanding the objectives of the study, and the full ability to communicate verbally in Spanish (the language used in the four universities).

### 2.3. Variables and Instruments

The socio-demographic variables collected were age, gender, academic year, living arrangement, dependent children, employment status, and route of access to university. Additionally, students’ perceived stress associated with various learning activities was assessed, including lectures, seminars/case studies, problem-based learning (PBL), simulation, group work, written assignments, clinical practicums, examinations, and oral presentations. Responses were recorded on a 4-point Likert scale, ranging from low stress “Not at all” and “A little” to high stress “Quite a bit” and “Very much”.

Sense of coherence (SOC) and its three dimensions—comprehensibility, manageability, and meaningfulness—were measured using the 13-item Orientation to Life Questionnaire (OLQ-13) [23], in its validated Spanish version [24]. This instrument evaluates the internal resources that enable individuals to solve problems when confronted with stressful situations throughout life [14]. The OLQ-13 comprises five items for comprehensibility, four items for manageability, and four items for meaningfulness. Responses are recorded on a 7-point Likert scale, ranging from “Never” and “Rarely” to “Very often” and “Always”. The OLQ-13 has demonstrated good internal consistency, with Cronbach’s alpha values between 0.70 and 0.92 [23,25,26], and retains the same psychometric properties as the original 29-item version. Total scores range from a minimum of 13 to a maximum of 91. For the manageability and meaningfulness subscales, a score of 4 indicates minimal capacity and 28 indicates maximal capacity; for comprehensibility, a score of 1 indicates minimal capacity and 35 indicates maximal capacity. In this study, the Cronbach’s alpha value was 0.841.

### 2.4. Data Collection

The final questionnaire was prepared by the principal investigator in collaboration with lead investigators from each participating university. The study proposal was presented to the deans of the participating faculties, who granted permission to access classrooms. Subsequently, at each university, the principal investigator and collaborating researchers distributed an information sheet outlining the study’s objectives, voluntary informed consent form, and the questionnaire to students during class. Students were informed that participation was voluntary, and that they could withdraw at any time without penalty. Data collection took place between December 2019 and March 2020, always outside examination periods and accounting for the differing academic calendars of each institution. No exclusion criteria were applied, aside from refusal to participate. To minimize potential sources of bias, participation was voluntary and anonymous, and all students received the same instructions during questionnaire administration. Data collectors were trained to ensure consistency, and incomplete responses were excluded from the final analysis.

### 2.5. Data Analysis

Descriptive statistics for quantitative variables are expressed as means and standard deviations, while qualitative variables are summarized using frequencies and percentages. Bivariate analyses were performed using Chi-squared tests for categorical variables and Student’s *t*-tests for continuous variables. Analysis of variance (ANOVA) with Tukey’s post hoc test was used for multiple mean comparisons. Statistical significance was set at *p* < 0.05. All analyses were conducted using SPSS version 29.0 for Windows (SPSS, Chicago, IL, USA).

### 2.6. Ethical Considerations

Participation was voluntary, and all responses were anonymized. The questionnaire included an introductory statement explaining the study’s purpose, intent, and data use. The study protocol (ID: CEBRU0015-2019; code: 07/2019) was approved by the Ethics and Biosafety Committee of the University of Girona. All procedures adhered to the principles of the 1964 Declaration of Helsinki and its later amendments, and all participants provided written informed consent.

## 3. Results

The questionnaire was administered to 1393 nursing students. Of these, 29 declined to participate and 63 were excluded due to missing data. Thus, the final sample consisted of 1301 students.

### 3.1. Sociodemographic Variables

The mean age of the participants was 22.37 years (SD = 4.42). Most participants were female, single, had no dependents, and were not employed. The primary route of university entry was via the standard university entrance examination, followed by vocational training. A detailed descriptive analysis of the sociodemographic variables is presented in Table 1.

### 3.2. Relation Between SOC and Sociodemographic Variables

The overall mean SOC-13 score was 62.65 (SD = 12.36; range: 27–90), while the mean subscale scores were 21.72 (SD = 5.64) for comprehensibility, 19.34 (SD = 4.70) for manageability, and 21.60 (SD = 3.93) for meaningfulness. Although the University of La Frontera in Chile had a slightly higher mean SOC score when compared to the three Spanish universities, this difference did not reach statistical significance (*p* = 0.470). Table 2 shows a comparison of the data by institution.

Bivariate analyses were performed to examine associations between SOC-13 scores and sociodemographic variables. Statistically significant differences were observed for age, marital status, academic year, route of university entry, and employment status, while no significant differences emerged for gender or having dependents. The post hoc Tukey test results indicated that students aged ≤20 years had significantly lower SOC-13 scores than those aged 21–24 years (*p* = 0.008), 25–28 years (*p* = 0.007), and ≥29 years (*p* ≤ 0.001). Significant differences in SOC-13 scores were also found between first- and third-year students (*p* = 0.004), first- and fourth-year students (*p* ≤ 0.001), second- and fourth-year students (*p* ≤ 0.001), and third- and fourth-year students (*p* = 0.004). Regarding living arrangement, married students differed significantly from single students (*p* ≤ 0.001) and those in common-law partnerships (*p* = 0.017). Students entering via the standard university entrance exam had higher SOC-13 scores than those coming from vocational training (*p* = 0.015) and those entering through the over-25 admission pathway (*p* = 0.001). Additionally, the comprehensibility and meaningfulness subscale scores were significantly related to gender. The detailed associations between SOC-13, its dimensions, and all sociodemographic variables are summarized in Table 3.

### 3.3. Relation Between SOC and Perceived Academic Stress

Regarding the perceived stress associated with teaching activities and assessments, the highest proportions of students reported high levels of stress for examinations, oral presentations, and written assignments. Conversely, lectures and seminars were correlated with low level of stress (Table 4).

When examining the relationships between SOC-13 and perceived stress, students who reported higher stress in lectures, seminars, PBL, group work, examinations, written assignments, and clinical practicums had significantly lower SOC-13 scores and lower scores in most SOC dimensions. However, perceived stress in simulation and oral presentations was not significantly associated with SOC-13 or its dimensions. Detailed comparisons are provided in Table 5.

## 4. Discussion

The findings of this study allowed us to address the primary aim: to explore the sense of coherence (SOC) in nursing students and to examine its relationships with sociodemographic variables and perceived stress associated with specific learning activities.

The sociodemographic profile of the participating nursing undergraduates was predominantly female, with a mean age of 22.65 years—characteristics that align closely with those reported in previous studies involving nursing students [27,28,29].

The mean SOC-13 score in our sample was 62.65 (SD = 12.36), a result comparable to that of Fernández-Martínez et al. [28], who reported a mean SOC-13 of 63.72 (SD = 9.86) in health sciences undergraduates. Although University of La Frontera in Chile obtained a higher SOC score compared to the three Spanish universities in our study, this difference did not reach statistical significance. Notably, these findings contrast with previous research. For example, nurses in Japan of Chinese origin obtained a mean SOC-13 of 57.7 [30], and Iranian university students scored 54.54 (SD = 6.46) [16]. Similarly, Gambetta-Tessini et al. [31] reported mean SOC-13 values of 55.56 and 58.01 among dental students in Australia, New Zealand, and Chile (*p* < 0.05), with Chilean students scoring lower.

To address potential cultural and educational influences on SOC and the perception of stress among university nursing students, it is important to consider the broader contextual differences between Spain and Chile. Although both countries share a common language and certain cultural traits, their educational systems differ in structure, pedagogical approaches, and institutional support mechanisms. For example, studies have highlighted that Latin American students may experience higher academic stress due to less institutional flexibility and fewer mental health resources [32]. In contrast, European higher education institutions—such as those in Spain—may offer more structured support systems, potentially fostering a stronger SOC. Moreover, cultural factors related to coping styles, social support, and perceived control may also contribute to differences in how students appraise and respond to stressors [33]. These contextual elements may partly explain the variations observed in SOC levels and stress perception between the two student groups.

In our sample, there were no statistically significant differences in total SOC-13 by gender. This contrasts with Mahammadzadeh et al. [26], who reported a significantly higher SOC in male university students (M = 9.81; SD = 11.83) versus female students (M = 56.30; SD = 12.97; *p* < 0.05).

When examining each SOC dimension, we observed that women scored higher than men in meaningfulness (women vs. men: 20.98 vs. 21.71; *p* = 0.014)—a finding similar to that reported by Colomer-Pérez et al. [34] (women vs. men: 21.02 vs. 19.02; *p* < 0.05). However, this result again contrasts with the study of Mahammadzadeh et al. [26], in which men outscored women (20.30 vs. 19.19) in meaningfulness. Conversely, male students in our study achieved higher scores in comprehensibility (22.84 vs. 21.51; *p* = 0.002) and slightly—but not significantly—higher scores in manageability (19.69 vs. 19.27; *p* = 0.286), in line with Mahammadzadeh et al. [26].

Significant differences emerged between age groups and total SOC (*p* ≤ 0.001), with SOC increasing with age: participants aged ≤20 years had a mean SOC of 60.55, whereas those aged ≥29 years scored 70.02. Similar trends have been documented elsewhere [27], with SOC rising up to age 30 and plateauing thereafter, as observed in Danish and Swedish populations [35,36,37].

Approximately 32% of our participants combined work and study—a marked decrease from the 57.2% reported in a previous study of Catalan nursing faculties [27]. Working students exhibited a significantly higher SOC than non-working peers (63.75 vs. 62.14; *p* = 0.029), consistent with Colomer-Pérez et al. [34] (59.12 vs. 55.51; *p* < 0.05). Salamonson et al. [38] found no significant difference in SOC between first-year nursing students in Sydney (*p* = 0.193), although their working students did trend toward slightly higher scores.

Marital status also influenced SOC: married (71.48) and divorced (69.29) students scored significantly higher than single students (62.21; *p* ≤ 0.001). A similar association between marital status and SOC has previously been reported in South Korean nursing students [39].

Regarding the relationships between SOC and perceived stress, higher stress in lectures (*p* = 0.002), seminars (*p* ≤ 0.001), PBL (*p* ≤ 0.001), group work (*p* ≤ 0.001), examinations (*p* = 0.010), written assignments (*p* ≤ 0.001), and clinical practicums (*p* ≤ 0.001) correlated with significantly lower SOC-13 scores and lower subscale scores. In contrast, stress perceived during simulation and oral presentations did not differ significantly across SOC levels. In all cases except for oral presentations and simulation, a higher SOC was associated with lower perceived stress. Comparable findings have been reported among dental students [31] and nursing students [39,40,41]. Stecz et al. [42], however, noted that medical students tend to experience stress during high-fidelity simulations but display substantial resilience, suggesting that factors beyond SOC may influence the perception of stress during simulation. Oral presentations and simulations have particular characteristics, such as immediacy, emotional intensity, and social exposure, which can favor the appearance of anxiety when speaking in public—a documented aspect in the field of health professionals [43]—and are very present in these types of teaching activities. It could be interpreted that not having well-developed competencies when speaking in public could limit the protective effect of SOC.

Finally, psychological resilience serves as a mediator between professional identity and academic burnout, suggesting that strengthening both professional identity and resilience may be an effective strategy for reducing academic exhaustion among nursing students [44]. Moreover, emotional intelligence may play a significant role in enhancing students’ sense of coherence (SOC), as higher emotional intelligence fosters empathy and promotes care that is grounded in a humanistic model—skills that are essential during clinical practice [45].

### 4.1. Limitations and Strengths

The primary limitation of this study is its cross-sectional design, which precludes causal inference. Additionally, the use of self-reported questionnaires may introduce response bias, as participants may have answered in a socially desirable manner. The sample, although multicenter in nature, was limited to nursing students from specific institutions in Spain and Chile, which may affect the generalizability of the findings to other contexts or countries. Future research should incorporate qualitative methods—such as in-depth interviews or focus groups—and mixed-methods approaches to obtain a more nuanced understanding of how SOC and stress interact over time.

A key strength is the study’s focus on a relevant issue in nursing education; in particular, the interplay between academic stress and SOC, which is considered a critical construct for mental health and adaptation to demanding academic contexts. By examining both sociodemographic variables and the impacts of specific learning activities, this study provides a more comprehensive perspective on factors influencing student well-being. These insights may inform targeted interventions to reduce academic stress among nursing students.

### 4.2. Practical Application and Future Research

Curricula and teaching methods should be adapted to include content and pedagogical strategies that foster resilience, positive coping, and SOC among nursing students. Early identification of students at risk for high stress and low SOC could facilitate the implementation of preventive and mental health-promotion programs within the university setting.

Future studies should evaluate interventions aimed at enhancing SOC in students with low baseline scores, in order to determine whether increasing SOC also reduces academic stress and improves performance in evaluations.

It will also be important to examine whether the implementation of resilience workshops or emotional intelligence training programs—both of which have been identified as protective factors against academic stress—contribute to strengthening the sense of coherence (SOC) among nursing students.

## 5. Conclusions

A higher sense of coherence was found to be associated with older age, advanced years of study, stable partner relationships, and engagement in paid employment during the considered nursing programs.

Participatory teaching activities—such as oral presentations, PBL, and written assignments—as well as examinations, were perceived as the most stressful by students.

Two SOC subscales showed differences related to gender: meaningfulness was higher in women, while comprehensibility was higher in men. Although global SOC was higher in men, we cannot definitively attribute this to gender alone.

In almost all academic activities (e.g., examinations, PBL, group work, seminars), higher SOC was correlated with lower perceived stress; however, no such relationship was observed for oral presentations and simulation. A strong SOC equips nursing students with better coping skills to manage academic stressors. Taken together, these results suggest that SOC functions as a protective factor against academic stress for most learning activities, although its influence is not uniform across all types of educational experiences.

## Figures and Tables

**Table 1:** Descriptive analysis of sociodemographic variables by sex (n = 1301).

	Women	Men	*p*
Gender n (%)	1094 (84.1)	207 (15.9)	
Mean age (SD) *	22.24 (4.43)	23.05 (4.32)	**0.015**
Grouped age n (%) **			**0.001**
≤20 years old	421 (38.5)	57 (27.5)	
21–24 years old	515 (47.1)	101(48.8)	
25–28 years old	92 (8.4)	31 (15.0)	
≥29 years old	66 (6.0)	18 (8.7)	
Cohabitation status n (%) **			0.885
Single	961 (87.8)	180 (87.0)	
Married	27 (2.5)	4 (1.9)	
Free union	100 (9.1)	22 (10.6)	
Divorced	6 (0.5)	1 (0.5)	
Course n (%) **			0.783
First	350 (32.0)	59 (28.5)	
Second	329 (30.1)	64 (30.9)	
Third	287 (26.2)	59 (28.5)	
Fourth	128 (11.7)	25 (12.1)	
University access route n (%) **			**0.008**
University entrance exam	834 (76.9)	140 (68.3)	
Another university degree	38 (3.5)	11 (5.4)	
Vocational training	180 (16.6)	42 (20.5)	
Access for those over 25 years old	25 (2.3)	12 (5.9)	
Access for people over 45 years old	8 (0.7)	0 (0)	
Work n (%) **			0.130
Yes	338 (30.9)	75 (36.2)	
No	756 (69.1)	132 (63.8)	
Dependents (n = 1094) n (%) **			0.179
Yes	19 (1.7)	1 (0.5)	
No	1075 (98.3)	206 (99.5)	

* Student’s *t*-test; ** Chi-squared test. Bold data indicate statistically significant differences.

**Table 2:** Analysis of the means of the overall SOC-13 and its subscales by university and by country of university (n = 1301).

	UdL M (SD)	URV M (SD)	UdG M (SD)	UFRO M (SD)	*p* *	Spain M (SD)	Chile M (SD)	*p* **
**SOC-13**	63.10 (12.72)	62.55 (10.95)	62.08 (12.50)	63.54 (15.49)	0.588	62.55 (11.94)	63.54 (15.49)	0.470
**Manageability**	19.52 (4.78)	19.25 (4.35)	19.27 (4.61)	19.40 (5.80)	0.872	19.33 (4.55)	19.40 (5.80)	0.875
**Comprehensibility**	21.84 (5.81)	21.74 (5.05)	21.38 (5.73)	22:30 (6:80)	0.395	21.65 (5.48)	22:30 (6:80)	0.283
**Meaningfulness **	21.73 (3.95)	21.56 (3.56)	21.43 (4.07)	21.84 (4.73)	0.661	21.57 (3.83)	21.84 (4.73)	0.513

UdL: University of Lleida; URV: Rovira i Virgili University; UdG: University of Girona; UFRO: University of La Frontera (Chile). * ANOVA; ** Student’s *t*-test.

**Table 3:** Relationships between SOC-13 scores and sociodemographic variables (n = 1301).

	SOC 13 M (SD)	*p*	Manageability M (SD)	*p*	Comprehensibility M (SD)	*p*	Meaningfulness M (SD)	*p*
Gender *								
Women	62.49 (12.32)	0.279	19.27 (4.71)	0.286	21.51 (5.62)	**0.002**	21.71 (3.88)	**0.014**
Men	63.51 (12.58)		19.69 (4.61)		22.84 (5.62)		20.98 (4.18)	
Grouped age **								
≤20 years	60.55 (12.48)	**≤0.001**	19.01 (4.70)	**0.001**	20.62 (5.63)	**≤0.001**	20.92 (4.13)	**≤0.001**
21–24 years old	62.91 (12.11)		19.28 (4.74)		21.87 (5.51)		21.76 (3.78)	
25–28 years old	64.50 (12.24)		19.69 (4.64)		22.65 (5.58)		22.16 (3.73)	
≥29 years old	70.02 (10.27)		21.15 (3.98)		25.46 (4.70)		23.40 (3.24)	
Cohabitation status **								
Single	62.21 (12.44)	**≤0.001**	19.26 (4.73)	**0.032**	21.52 (5.63)	**≤0.001**	21.43 (3.98)	**≤0.001**
Married	71.48 (10.17)		21.65 (4.13)		26.03 (4.85)		23.81 (2.70)	
Free union	64.19 (11.30)		19.43 (4.49)		22.28 (5.46)		22.48 (3.58)	
Divorced	69.29 (8.52)		21.00 (3.05)		24.86 (4.49)		23.43 (2.15)	
Course **								
First	60.73 (12.40)	**≤0.001**	18.88 (4.85)	**≤0.001**	20.98 (5.62)	**≤0.001**	20.87 (3.95)	**≤0.001**
Second	61.70 (11.67)		19.22 (4.44)		21.21 (5.38)		21.27 (3.99)	
Third	63.75 (12.40)		19.38 (4.73)		22.13 (5.62)		22.23 (3.82)	
Room	67.78 (12.37)		20.78 (4.60)		24.07 (5.72)		22.93 (3.44)	
University access route (n = 974) **								
University entrance exam	61.80 (12.57)	**≤0.001**	19.16 (4.80)	0.053	21.28 (5.66)	**≤0.001**	21.35 (4.01)	**0.002**
Another university degree	62.98 (11.47)		19.08 (4.43)		21.86 (5.43)		22.04 (3.49)	
Vocational training	64.66 (11.30)		19.72 (4.32)		22.70 (5.24)		22.24 (3.75)	
Access for those over 25 years old	69.86 (11.25)		21.24 (4.45)		25.43 (5.36)		23.19 (3.07)	
Access for people over 45 years old	69.50 (4.41)		20.25 (2.19)		26.50 (3.25)		22.75 (1.91)	
Work *								
Yes	63.75 (12.60)	**0.029**	19.60 (4.69)	0.167	22.08 (5.87)	0.117	22.07 (3.89)	**0.003**
No	62.14 (12.22)		19.22 (4.70)		21.55 (5.52)		21.38 (3.94)	
Dependents (n = 1094) *								
Yes	64.30 (11.06)	0.549	19.65 (3.47)	0.765	22.70 (6.42)	0.433	21.95 (2.96)	0.685
No	62.63 (12.38)		19.33 (4.71)		21.70 (5.62)		21.59 (3.95)	

* Student’s *t*-test; ** ANOVA. Bold data indicate statistically significant differences.

**Table 4:** Level of stress perceived by students according to academic activity (n = 1301).

	Low Stress n (%)	High Stress n (%)
Lectures	1048 (80.7)	251 (19.3)
Seminar/case studies	738 (57.1)	555 (42.9)
Simulation	604 (47.1)	679 (52,9)
Group work	610 (46.9)	690 (53.1)
Clinical practicums	571 (45.8)	675 (54.2)
PBL	457 (37.5)	762 (62.5)
Written assignments	462 (35.6)	837 (64.4)
Oral presentations	262 (20.2)	1037 (79.8)
Examinations	86 (6.6)	1212 (93.4)

**Table 5:** Relationships between the perception of stress according to academic activities and the SOC-13.

	SOC 13 M (SD)	*p*	Manageability M (SD)	*p*	Comprehensibility M (SD)	*p*	Meaningfulness M (SD)	*p*
Lectures (n = 1299)								
Low stress	63.20 (12.29)	**0.002**	19.55 (4.68)	**0.002**	21.92 (5.65)	**0.010**	21.73 (3.88)	**0.014**
High stress	60.48 (12.43)		18.51 (4.66)		20.91 (5.52)		21.06 (4.13)	
Seminar (n = 1293)								
Low stress	64.22 (11.58)	**≤0.001**	19.98 (4.45)	**≤0.001**	22.31 (5.43)	**≤0.001**	21.92 (3.68)	**0.001**
High stress	60.61 (13.08)		18.50 (4.86)		20.91 (5.83)		21.19 (4.21)	
PBL (n = 1219)								
Low stress	65.11 (11.84)	**≤0.001**	20.26 (4.50)	**≤0.001**	22.75 (5.48)	**≤0.001**	22.09 (3.77)	**0.003**
High stress	61.57 (12.40)		18.91 (4.67)		21.26 (5.62)		21.40 (4.02)	
Group work (n = 1300)								
Low stress	64.18 (12.24)	**≤0.001**	20.02 (4.59)	**≤0.001**	22.33 (5.60)	**≤0.001**	21.83 (3.82)	**0.045**
High stress	61.31 (12.33)		18.74 (4.71)		21.18 (5.62)		21.39 (4.02)	
Exam (n = 1299)								
Low stress	65.98 (13.10)	**0.010**	20.60 (5.01)	**0.010**	23.71 (5.78)	**0.001**	21.66 (4.33)	0.883
High stress	62.43 (12.27)		19.25 (4.66)		21.58 (5.60)		21.60 (3.90)	
Written work (n = 1299)								
Low stress	64.70 (12.49)	**≤0.001**	20.02 (4.66)	**≤0.001**	22.57 (5.71)	**≤0.001**	22.11 (3.92)	**0.001**
High stress	61.53 (12.16)		18.97 (4.68)		21.24 (5.55)		21.32 (3.91)	
Clinical practicums (n = 1246)								
Low stress	64.45 (11.83)	**≤0.001**	19.94 (4.55)	**≤0.001**	22.57 (5.49)	**≤0.001**	21.95 (3.75)	**0.009**
High stress	61.37 (12.56)		18.92 (4.74)		21.09 (5.66)		21.36 (4.08)	
Simulation (n = 1283)								
Low stress	63.23 (12.05)	0.158	19.61 (4.57)	0.065	22.03 (5.54)	0.080	21.58 (3.87)	0.776
High stress	62.26 (12.56)		19.13 (4.78)		21.48 (5.70)		21.65 (3.96)	
Oral presentation (n = 1299)								
Low stress	63.12 (12.92)	0.498	19.45 (4.74)	0.656	21.93 (6.00)	0.513	21.73 (4.14)	0.538
High stress	62.54 (12.23)		19.31 (4.69)		21.66 (5.55)		21.57 (3.88)	

Student’s *t*-test. Bold data indicate statistically significant differences.

## Data Availability

Data will be made available on request.

## Supplementary Materials

The following supporting information can be downloaded at: https://www.mdpi.com/article/10.3390/nursrep15080288/s1, Table S1: STROBE checklist for cross-sectional studies.

## Public Involvement Statement

No public involvement in any aspect of this research.

## Guidelines and Standards Statement

This manuscript was drafted against the STROBE (Strengthening the Reporting of Observational Studies in Epidemiology) guidelines for reporting observational studies involving cross-sectional research.

## Abbreviations

The following abbreviations are used in this manuscript:

PBL	Problem-based learning
SOC	Sense of coherence
UdG	University of Girona (Spain)
UdL	University of Lleida (Spain)
UFRO	University of La Frontera (Chile)
URV	University of Rovira i Virgili (Spain)
